# A numerical simulation of air flow in the human respiratory system for various environmental conditions

**DOI:** 10.1186/s12976-020-00133-8

**Published:** 2021-01-06

**Authors:** Alibek Issakhov, Yeldos Zhandaulet, Aizhan Abylkassymova, Assylbek Issakhov

**Affiliations:** 1 Al-Farabi Kazakh National University, av. al-Farabi 71, 050040 Almaty, Republic of Kazakhstan; 2 Kazakh British Technical University, Almaty, Republic of Kazakhstan

**Keywords:** Air flow in the human respiratory system, Alveolar state, Heat transfer in the nasal cavity, Navier, Stokes equation, Finite volume method

## Abstract

The functions of the nasal cavity are very important for maintaining the internal environment of the lungs since the inner walls of the nasal cavity control the temperature and saturation of the inhaled air with water vapor until the nasopharynx is reached. In this paper, three-dimensional computational studies of airflow transport in the models of the nasal cavity were carried out for the usual inspiratory velocity in various environmental conditions. Three-dimensional numerical results are compared with experimental data and calculations of other authors. Numerical results show that during normal breathing, the human nose copes with heat and relative moisture metabolism in order to balance the intra-alveolar conditions. It is also shown in this paper that a normal nose can maintain balance even in extreme conditions, for example, in cold and hot weather. The nasal cavity accelerates heat transfer by narrowing the air passages and swirls from the nasal concha walls of the inner cavity.

## Background

The human nasal cavity acts as an important component of the respiratory system with many vital functions, including heating, filtering, moisturizing the air flow and smell. These functions are based on transport phenomena, which depend on the nature of the air flow in the nasal structure.

After the first studies of nasal function [5, 12, 13, 43], it was clear that inhaling through the nasal cavity causes the incoming ambient air to become almost alveolar, then is completely saturated with water vapor and heated to a person’s body temperature by the time it reaches the pharynx. These results were confirmed in many studies that collected data on air temperature values from various places in the upper respiratory tract throughout the respiratory cycle. Nevertheless, in [32], it was noted that these statements are true for calm (uniform) breathing, and for some cases, at high speeds in the intrathoracic airways, it is necessary to carry out additional conditioning to fully prepare the inhaled air to alveolar conditions. However, the exact characteristics and distribution of transport phenomena are still unknown even for normal or healthy breathing.

Depending on the local Reynolds numbers (Re) for different structures of the nasal air flow, they can undergo various flow regimes (laminar, transitional and turbulent). The normal rate of respiration in the nasal cavity is seen as a laminar flow, which accelerates in certain places of the nose and turns into a transient flow, gradually turning into a turbulent flow due to a decrease in cross-sectional area [6]. In the paper [10], measurements were made using laser anemometry on a human nose model and found that the flow field is mainly laminar and the velocity profiles are almost parabolic in all cross sections.

Turbulent flow usually occurs at Re > 2000, but depending on the complexity of the airways, the transition to a turbulent regime can occur at lower values of the Re number (below the value of 2000). Turbulent flow leads to better mixing of heat and moisture, which contributes to the functions of the nasal cavity. For adults, airflow rates can vary from 80 to 200 ml/s with calm breathing and from 200 to 1000 ml/s during exercise [1, 37], with a Re range from a few hundred to several thousand. However, in [7] at flow rates of 115–170 ml/s, irregular flow in the nasal cavity was revealed, which makes the assumption of laminar flow controversial for calm breathing.

With the development of high-performance computing in recent years, many researchers have considered simulation using computational fluid dynamics (CFD) as an alternative approach to studying airflow in human respiratory systems. Compared to natural measurements, CFD modeling is advantageous for a detailed study of air flow in the nasal cavity model. However, an accurate airflow forecast requires careful selection of CFD models and rigorous testing. In papers [4, 26, 40], a laminar model is used to simulate airflow in the nasal cavity at relatively low speeds, when the kinetic energy of turbulence is insignificant.

In [14, 33, 34], modeling of unsteady two-dimensional and three-dimensional models is considered to study the transfer of air flow in the nasal cavity of a person and its general ability to condition air. The results of these works showed that the nose can effectively provide about 90% of the heat and water flows needed to condition the surrounding respiratory air, bringing it to alveolar conditions in various environmental conditions. A 3D anatomical copy of the human nose showed the best results and was able to provide 92% of the heat and 96% of the moisture needed to condition the inhaled air. However, it should be noted that in [33] two-dimensional geometry was considered as sections of the nasal cavity, and in [34] the three-dimensional model was constructed in a rough form and shaped like a trapezoid. It should be noted that during the simulation, simplifications in geometry can lead to uncertainties or inaccuracies in the numerical results compared with real nasal cavities.

The accuracy of the simulation strongly depends on the choice of the numerical method and rigorous verification with experimental data. Several tests of numerical CFD models of airflow in the human nasal cavity were performed, which compared the pressure drop in the nasal cavity and the static pressure on the walls of the nose [4, 31, 42]. More detailed comparisons of various characteristics were also made in [2, 3, 8, 45], however, these calculations were performed for not specified nasal cavities. Most of these previous studies have tested their models on a limited range of flow rates. In [28], the accuracy of various CFD models was estimated for modeling airflow velocities under various respiration conditions in the right nasal cavity, based on computed tomography (CT).

Some investigation in the past has been limited by the poor presentation of complex nasal geometry, lack of detailed airflow comparisons, and limited computing power. The high computational cost limits the use of detailed geometry; therefore, many authors conducted very limited studies for the simplified geometry of the human nasal cavity [27, 38, 41, 44].

There are a few numerical studies that consider heat and moisture transfer. So in the papers [29, 30], it is assumed that the walls of the nasal cavity representing the nasal mucosa have a constant temperature. In [36], air conditioning, heating and humidification of the air in the nasal cavity are considered. This paper presents experimental and computational results that were aimed at promoting modern physiological research and practical medicine related to the health of the respiratory system. Also [9, 35, 39, 41, 44] give estimates of the nose morphology in relation to anomalies and diseases using three-dimensional computational models by providing doctors with the understanding, necessary to make informed decisions regarding surgical interventions.

The aim of this work is to study the various properties of the flow as heating and moisturizing using computational methods on an anatomically accurate model of the nose. Detailed velocity analysis is presented and compared with data from the measurement [11] and computational data [28]. The abilities of the nasal cavity were studied: heating and moisturizing the air during normal breathing in different environmental conditions. To study the flow in the nasal cavity, the ANSYS Fluent was used.

## Materials and methods

### The mathematical model

To simulate the air flow in the nasal cavity, the basic equations for the conservation of mass, momentum, temperature and relative humidity are determined as follows:

1 $$ \nabla \cdot U=0 $$

2 $$ \frac{\partial U}{\partial t}+\left(U\cdot \nabla \right)U=-\frac{1}{\rho}\nabla P+\vartheta {\nabla}^2U $$

3 $$ \frac{\partial T}{\partial t}+\left(U\cdot \nabla \right)T=\frac{k}{\rho {c}_p}{\nabla}^2T $$

4 $$ \frac{\partial C}{\partial t}+\left(U\cdot \nabla \right)C=D{\nabla}^2\mathrm{C} $$

where U is the velocity vector, t is the real time, P is the flow pressure, C is the concentration of water vapor, c_р_ is the specific heat, D is the molecular diffusion coefficient, T is the temperature, *ρ*, *ϑ* and k are the density, kinematic viscosity and thermal conductivity, respectively.

The ANSYS Fluent was used to investigate the flow in the nasal cavity. For numerical study, second-order spatial discretization schemes for pressure and momentum were used. Thus, the real problem will be solved in a discrete form by applying the finite volume method. The SIMPLE algorithm was used to communicate between pressure and velocity. The SIMPLE algorithm can be represented as the following sequence of steps:

1. Representations of the initial pressure field *P*^0^ and set *P*^∗^ = *P*^0^, *t* = 0.
2. Definitions of the initial velocity field *u*^0^, *v*^0^, *w*^0^.
3. The solution of the equations of motion.
4. The solution of the equation for *P*' and *P* calculation by adding *P*' to *P*^∗^.
5. Using velocity correction formulas.
6. If there was ∣*P* ' ∣ little in all nodes of the computational grid, then it was assumed *P*^0^ = *P*, *u*^0^ = *u*, *v*^0^ = *v*, *w*^0^ = *w*, *t* = *t* + *Δt*. Otherwise, it was used the result *P* as *P*^∗^ and it was proceeded to stage 3.
7. If *t* < *T*_max_, then we have a return to step 3.

All details of applying this SIMPLE algorithm can be found in papers [15–25, 46].

## Methods

### Verification of the model

The full three-dimensional structure of the nasal cavity provides a very complex path of air passage. To provide comprehensive studies of the transport mechanisms of the nasal cavity, three-dimensional test problem has been done. For all calculations, it is assumed that the walls of the nasal cavity and nasal concha are motionlessly solid. The airflow in the nasal cavity is laminar and incompressible due to low speeds. The walls of the nasal cavity are considered to be completely saturated with water vapor and the temperature near the body due to the wet mucous layer reaches the vascular vessels of the nasal wall.

### Three-dimensional numerical simulation

To bring the numerical results closer to the real problem, it will need to use three-dimensional models of the nasal cavity, since heating and humidification of the inhaled air strongly depends on the structure of the nasal cavity walls. Therefore, computational models should include more realistic three-dimensional geometry descriptions in order to determine the effect of complex geometry on various characteristics such as velocity, heat and mass transfer, and relative humidity.

**Fig. 1:**
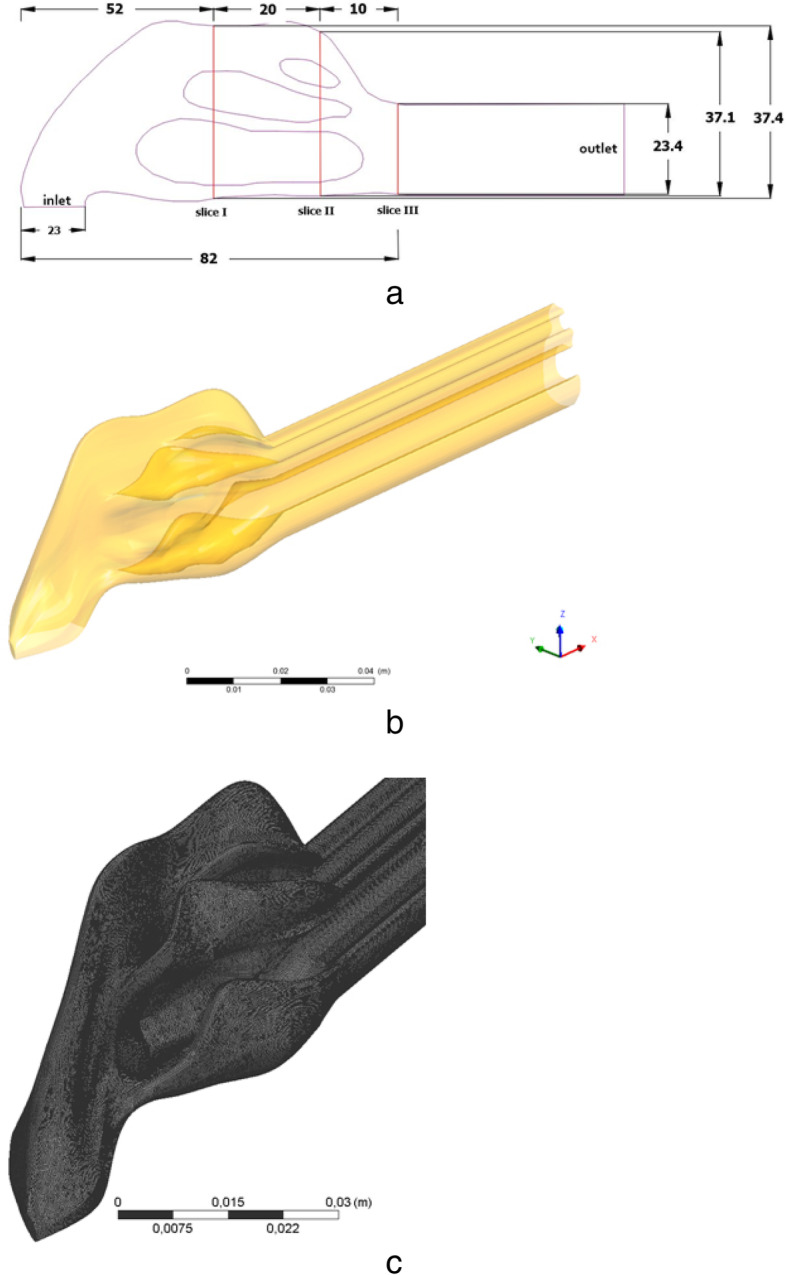
**a**-**b** Physical geometry of the studied area (all dimensions in mm), **c** the computational grid of a complex nasal cavity of a person.

In the paper [11], data from the measurements were made of the air flow in a nose prototype using a hot wire anemometer in a large-scale physical model of the nasal cavity, based on computed tomography of the right nasal cavity of a healthy man. In the measurements, various respiration rates were used, equivalent to 180 ml/s, 560 ml/s and 1100 ml/s in a real human nose, which corresponds to calm breathing, medium inhalation and intensive inhalation, respectively. Fig. 1 a, b displays the measurement setup and the relative position of the measurement sites on three slices, which were used to verify the obtained numerical values.

The numerical model included the area from the front tip of the nose to the posterior end of the nasal concha. As displayed in Fig. 1a, b, the nasopharynx was expanded to fit the experimental setup.

**Fig. 2:**
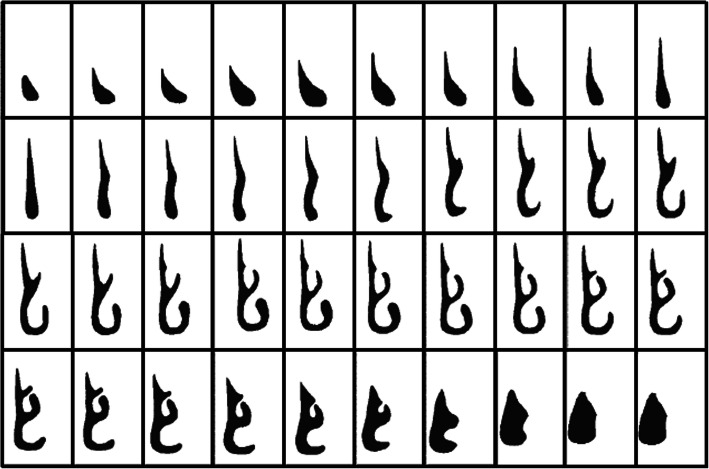
The 2D digitized cross-sections of 40 computed tomographic (CT) images of the respiratory tract of a healthy man

**Fig. 3:**
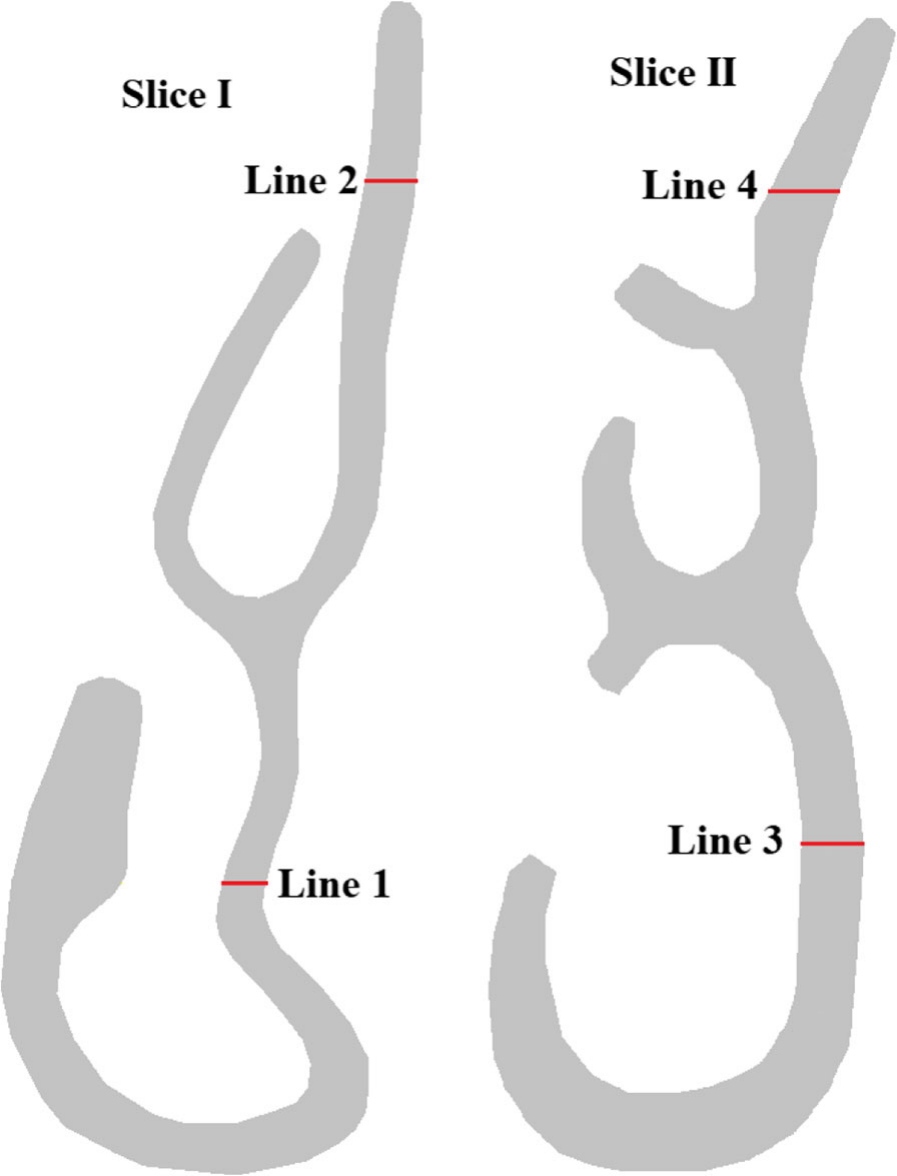
Location of lines 1–4 at slices 1 and 2

The geometry of the human nasal cavity was created by aligning and processing 40 computed tomographic (CT) images of the respiratory tract of a healthy man. Using the AutoCAD software, intermediate geometric shapes of the nasal cavity were created that correspond to the average physical parameters of the human nasal cavity. From these idealized 2-D images (Fig. 2), a 3-D complex human nasal cavity was created. The locations of the anemometers in the study area are presented in Fig. 3.

**Fig. 4:**
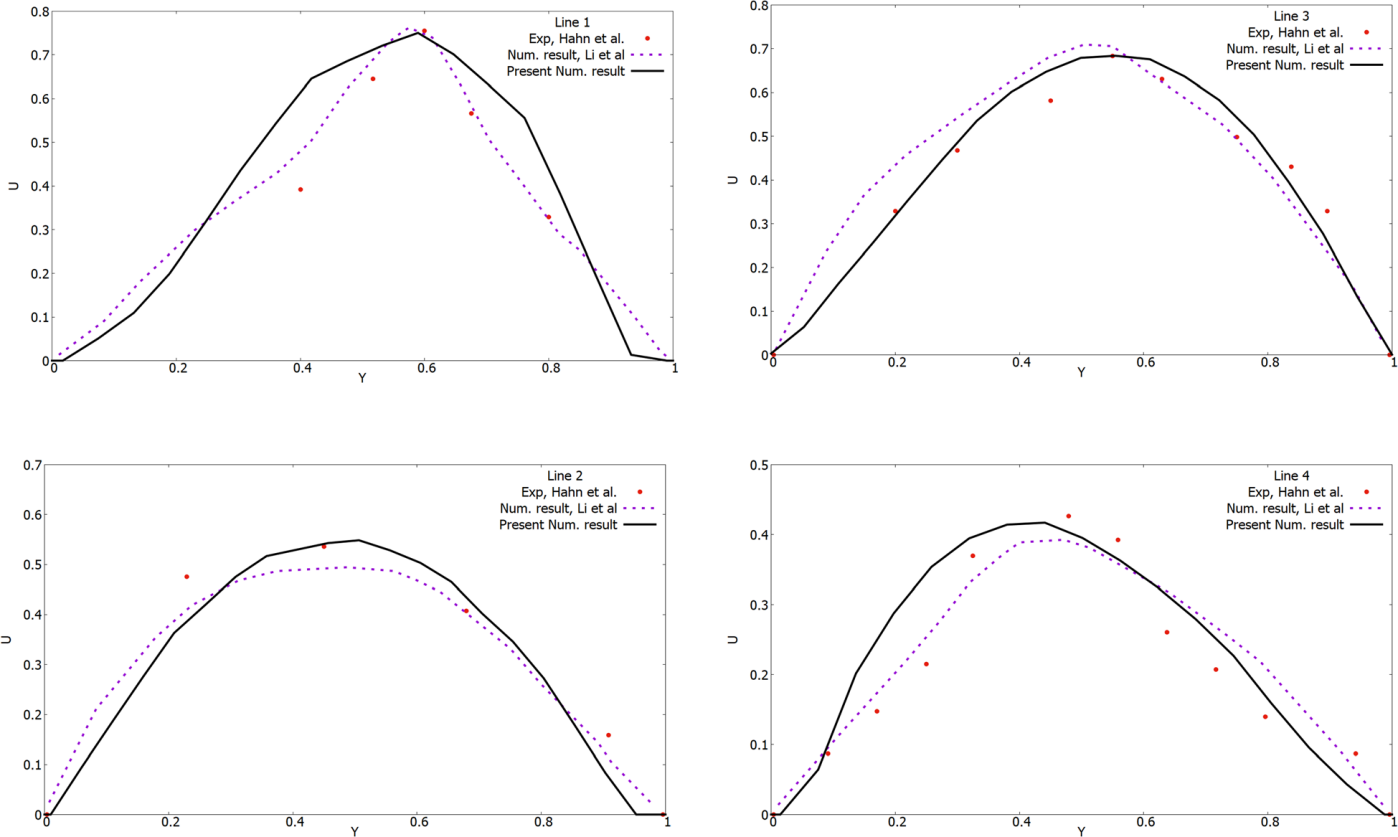
Comparison of the profiles of the horizontal velocity (U) component on lines 1–4 with the numerical results of other authors [28] and experimental data [11]

As can be seen from Fig. 3, in slices 1 and 2, 4 lines were located for measuring velocity at these points in the section. The computational grid of the studied area is presented in Fig. 1c. The final computational grid of the nasal cavity consisted of 6,876,463 elements (Fig. 1c). Comparisons of velocity profiles with measurement values [11] on lines 1–4 are presented in Fig. 4.

**Fig. 5:**
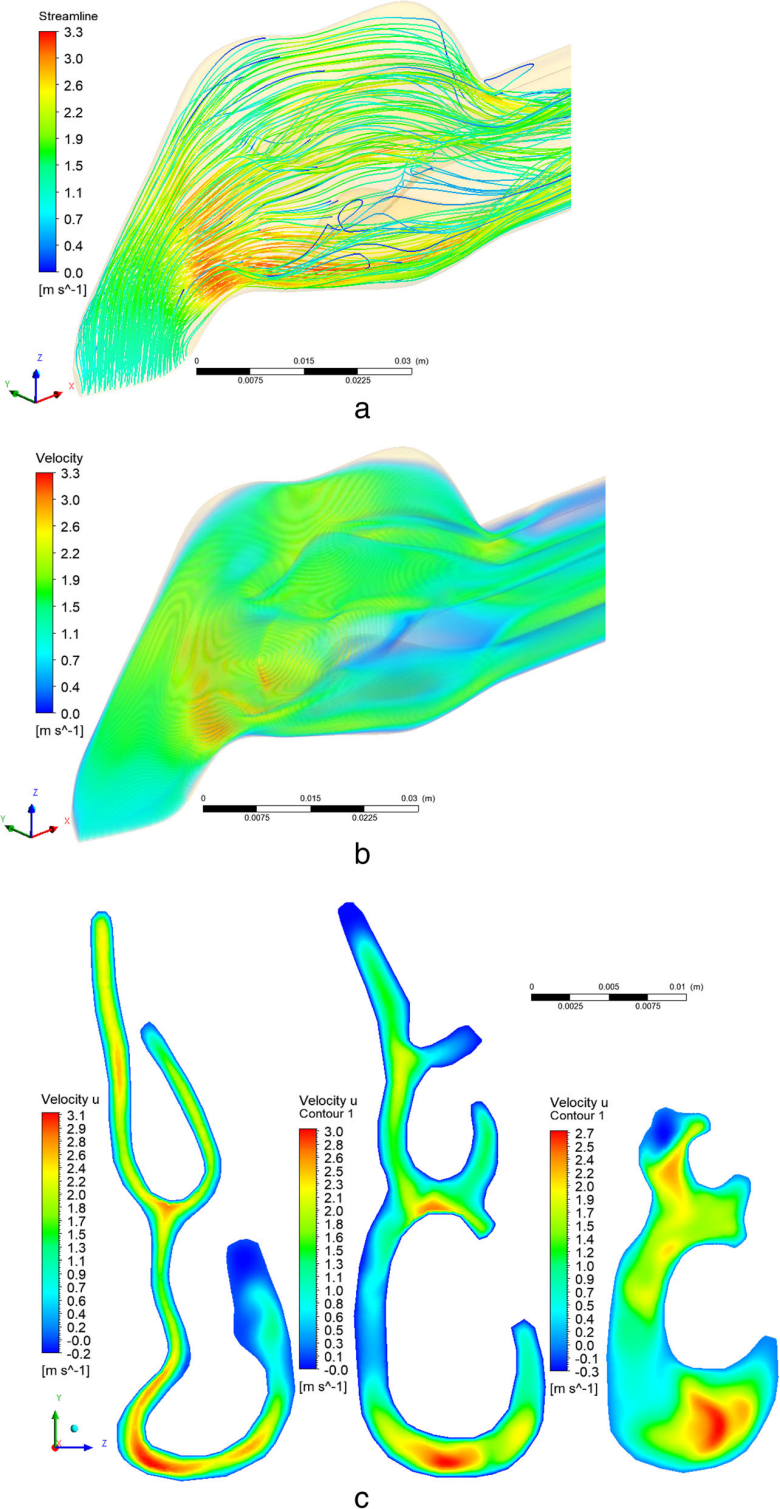
The longitudinal components of the air flow rate in the nasal cavity. **a**-**b** three-dimensional distributions of the longitudinal components of the air flow rate with the deposition of streamlines, **c** the two-dimensional contours of the longitudinal components of the velocity of the cross section 1–3

The velocity profiles were dimensionless by the value of the local maximum velocity. The X-axis for Y was dimensionless by the value of the local maximum distance. As shown in Fig. 4, the forecasts of the laminar model are in good agreement with measurement values [11]. The directions of the air flow in the nasal cavity and two-dimensional contours in sections 1–3 are shown in Figs. 5a, b and c. As expected, in the narrow channels of the nasal cavity the air flow accelerated and reached a maximum value of 3.33 m/s (Fig. 5a, b). It can be also noticed that due to the deep shells of the nasal cavity, vortices arise, which subsequently can well affect the heat and mass transfer. The two-dimensional velocity contours presented in Fig. 5c confirm the allegations of air flow acceleration due to the narrowing of the nasal concha and, as a result, the occurrence of vortices.

Despite some unevenness in the flow field profiles, it can be noted that the proposed model is in good agreement with the measurement values [11] under conditions of calm breathing. It can also be noted that the numerical results obtained show much closer values to the measurement data [11] than the obtained computational values in the paper [28]. However, the inaccuracies obtained in this work can be explained by the fact that the subtle features of the nasal cavity cannot be accurately measured due to the limited resolution of existing imaging methods. Accordingly, a model similar to the nasal cavity was developed (Fig. 1), in which the dimensions are taken from the averaged data on the human nasal cavities [11]. The model used allows us to comprehensively study a large number of the nasal cavity functions with respect to the structural components of the nasal cavity and the corresponding heat and mass transfer. It should be noted that in real conditions, the nose walls can be unequal or equal to the alveolar conditions, especially during exercise, and future models should clarify these assumptions.

## Results

### Numerical study of heating and moisturizing the air in the human nose

The air flow in the nasal cavity of a person plays an important role in many physiological functions of the nose, such as heating and moisturizing the flow of air and others. In this section, the proposed model is used to predict air flow and related transport phenomena in human nasal cavities.

It is assumed that the flow of heat and water vapor is released from the inside of the nasal mucosa. Normal respiration was chosen as a reference base, and then the effect of changes in ambient temperature was investigated. This study serves as the basis for a better understanding of transport phenomena in the nasal cavity (heat, mass), which are the main functions of the nose.

**Fig. 6:**
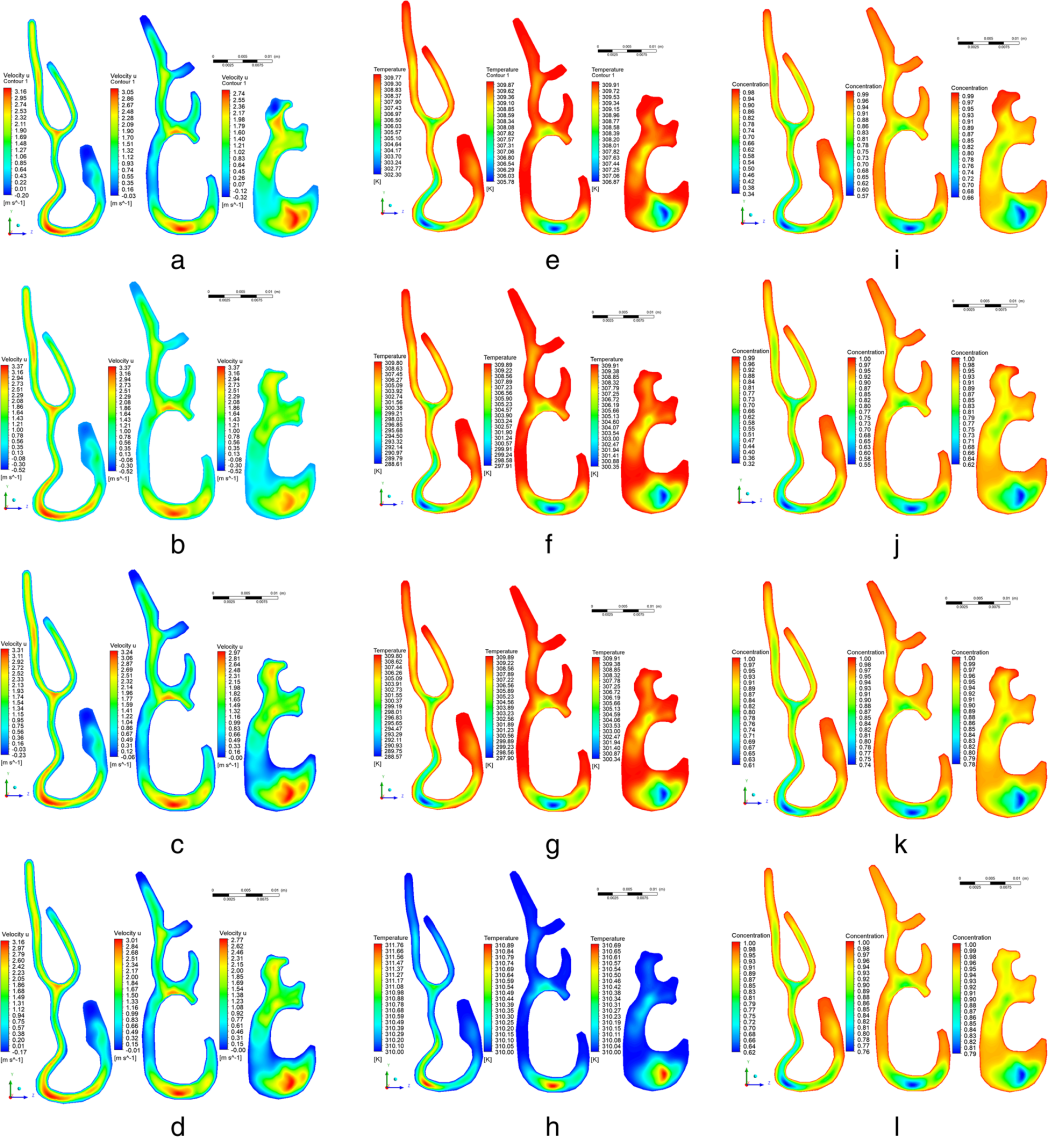
Two-dimensional and three-dimensional distributions of the longitudinal components of the flow rate for different environmental conditions: **a**-**b** at an ambient temperature of 25°C and humidity on the walls of the nasal cavity of 20% for different sections (sections 1-3). **c**-**d** at an ambient temperature of 5°C and humidity on the walls of the nasal cavity of 20% for different sections (sections 1-3). **e**-**f** at an ambient temperature of 5°C and humidity on the walls of the nasal cavity of 90% for different sections (sections 1-3). **g**-**h** at an ambient temperature of 40°C and humidity on the walls of the nasal cavity of 90% for different sections (sections 1-3). **i**-**j** at an ambient temperature of 25°C and humidity on the walls of the nasal cavity of 20% for different sections (sections 1-3). **k**-**l** at an ambient temperature of 5°C and humidity on the walls of the nasal cavity of 20% for different sections (sections 1-3). **m**-**n** at an ambient temperature of 5°C and humidity on the walls of the nasal cavity of 90% for different sections (sections 1-3). **o**-**p** at an ambient temperature of 40°C and humidity on the walls of the nasal cavity of 90% for different sections (sections 1-3). **q**-**r** at an ambient temperature of 25°C and humidity on the walls of the nasal cavity of 20% for different sections (sections 1-3). **s**-**t** at an ambient temperature of 5°C and humidity on the walls of the nasal cavity of 20% for different sections (sections 1-3). **u**-**v** at an ambient temperature of 5°C and humidity on the walls of the nasal cavity of 90% for different sections (sections 1-3). **w**-**x** at an ambient temperature of 40°C and humidity on the walls of the nasal cavity of 90% for different sections (sections 1-3).

The studied area was identical to the second test problem of Fig. 1. It is believed that the walls of the nasal cavity are completely saturated with water vapor and due to the wet mucous layer and rich underlying vascular bed, the temperature values are close to body temperature. The temperature on the nose walls is taken equal to 37 °C, the humidity on the walls is taken 100%. Environmental conditions were taken as in paper [33], the temperature of the inhaled air is 25 °C and the relative humidity is 20%. Figure 6 shows the two-dimensional and three-dimensional distributions of the longitudinal components of the flow velocity for various sections (sections 1–3) with these conditions. From the obtained results, it can be noted that the global behavior of the air flow was not changed, but, however, the maximum longitudinal velocity increased to 3.47 m/s (Fig. 6a). The increase in maximum longitudinal velocity was affected by the conditions of heating and moisturizing on the walls of the nasal cavity. Figure e shows the process of heating inhaled air for various sections. From the results of Fig. 6e, it can be noticed that the inhaled air in section 3 is heated, and the air temperature is varying between 34 and 37 °C. The concentration of water vapor in the 3 section reaches the value of 0.66–0.99 (Fig. 6i). This structure of the nasal cavity increases the local rate of heat and moisture transfer due to the narrowing of the nasal channels for air. This constriction in the nasal cavities leads to the appearance of eddies downstream.

To study the effect of respiration at various temperature and humidity environments, several simulations were carried out using the proposed model. Three modes were chosen for modeling heat and mass transfer in the nasal cavity for normal inspiration in extreme environments: at an ambient temperature of 40 °C and humidity on the walls of the nasal cavity 90%, at ambient temperature 5 °C and humidity on the walls of the nasal cavity 20%, ambient temperature 5 °C and humidity on the walls of the nasal cavity 90%. The velocity of air flow in the nasal cavity was used the same in all cases, however, the transfer process varies depending on the conditions. Figs. 6b, f, j show the simulation values at an ambient temperature of 5 °C and humidity on the walls of the nasal cavity of 20%. As shown in Fig. 6b, at a temperature of 5 °C and a humidity of 20%, the maximum velocity reached 3.56 m/s. The temperature of the inhaled air in section 3 ranges from 28 to 37 °C. However, it should be noted that a temperature of 28 °C occurs only in a small area, with an average temperature of 35 °C. As shown in Fig. 6j, inhaled air with a moisture content of 20% is moistened with water vapor from 0.62–0.99 until the nasopharynx is reached. In general, water vapor distributions are the same as the results from Fig. 6a.

Figure 6c shows the results of numerical modeling at an ambient temperature of inhaled air of 5 °C with humidity on the walls of the nasal cavity of 90%. The flow behavior at a temperature of 5 °C and a humidity of 90% is the same as the results of Fig. 6c. The results of Fig. 6 g and 6f are the same, since the conditions for the temperature of the inhaled air are the same. From the results of Fig. 6 k it can be seen that the concentration of inhaled air in section 3 is in the range 0.78–1.0. Figs. 6d, 6 h, 6 l show the results of numerical modeling at an ambient temperature of inhaled air of 40 °C with a nasal cavity humidity of 90%. At a temperature of inhaled air of 40 °C and with a humidity of 90%, the maximum flow velocity reaches a value of 3.4 m/s. The nasal cavity not only heats the inhaled air, but in some critical cases it can also cool. As shown in Fig. 6 h, inhaled air at 40 °C is cooled to body temperature. The concentration of inhaled air when passing through cross section 3 is 0.79–1.0, which is about an alveolar condition.

**Fig. 7:**
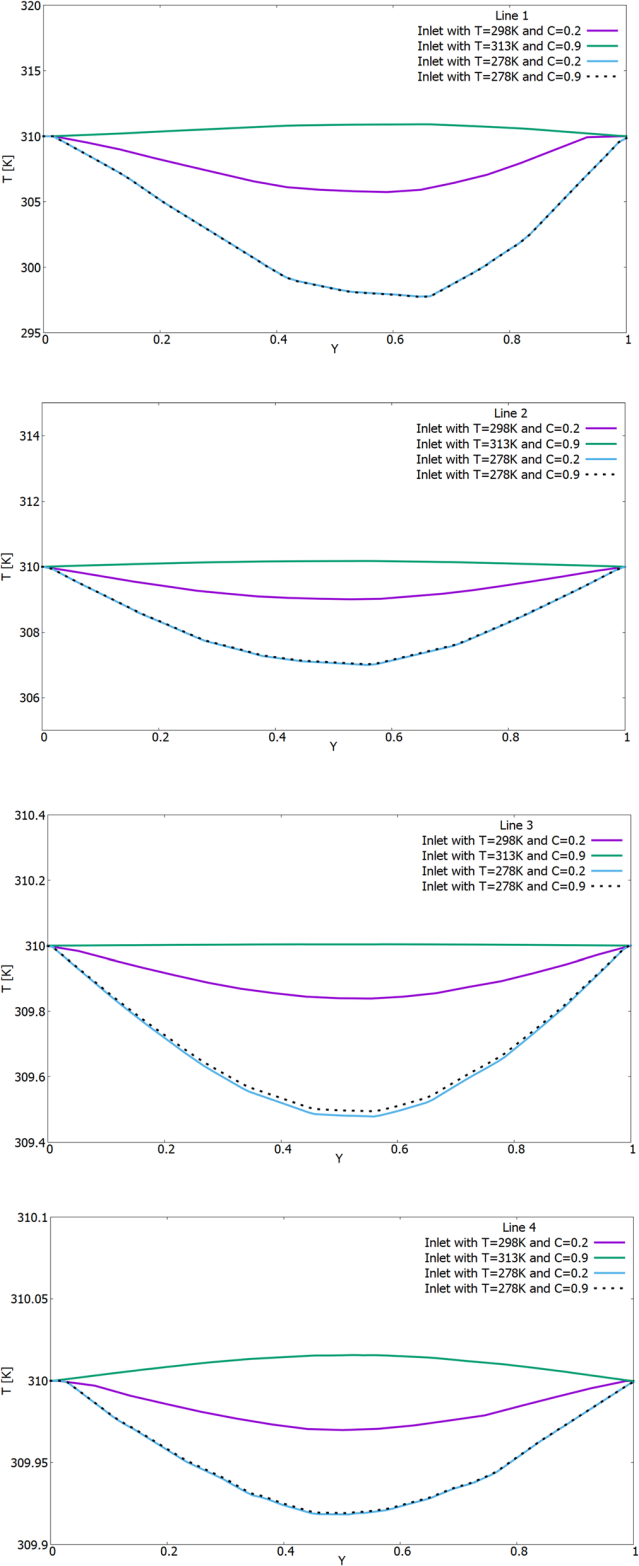
Comparison of temperature profiles (T) on lines 1–4 under different environmental conditions (for different ambient temperature and humidity on the walls of the nasal cavity: 1) 25 °C and 20%, 2) 5 °C and 20%, 3) 5 °C and 90%, 4) 40 °C and 90%)

**Fig. 8:**
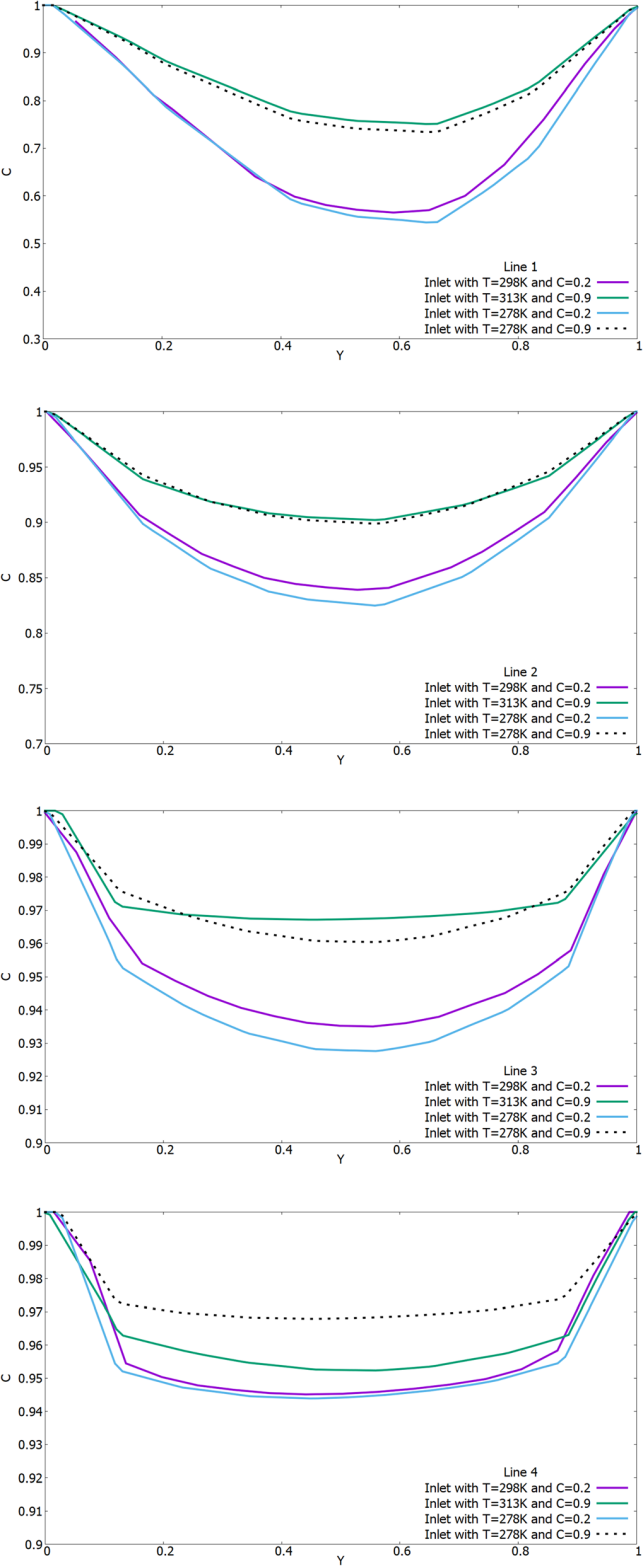
Comparison of concentration profiles on lines 1–4 under different environmental conditions

From the above results, it can be concluded that the nasal cavity balances the inhaled air with the internal conditions of the body with remarkable efficiency, and is almost independent of the surrounding air state. As a comparison, the influence of external conditions in Figs. 7 and 8 shows the temperature and concentration profiles on lines 1–4 from section 1–2 (Fig. 3). The X-axis for Y was dimensionless by the value of the local maximum distance. And the concentration on all the obtained numerical results is indicated as dimensionless, since it shows the mass fraction.

The results of Fig. 7 show the difference in temperature distribution on measuring lines 1–4. In all the results, it can be seen that the temperature of the inhaled air tends to the conditions of the walls of the nasal cavity, that is, to the body temperature of 37 °C. As shown in Fig. 8, the concentration profiles on the measuring lines are the same under the same humidity conditions of the inhaled air. Like temperature values, the concentration of water vapor also tends to the conditions of the walls of the nasal cavity, that is, to a concentration equal to 1.0.

In this work, a realistic geometry of the human nasal cavity was used, but, however, it must be taken into account that this geometry is not universal since each person has his own structure of the nasal concha. However, the created three-dimensional geometry of the human nasal cavity can be of great benefit as a standard nasal replica for testing various conditions of ambient temperature, relative humidity and the rate of inhaled air flow.

## Conclusion

This study is a comprehensive work on modeling the processes of heat and mass transfer in a 3D model of the human nasal cavity. The purpose of this study was to use the proposed computational model to investigate the dynamic ability of the nasal cavity to heat and moisten the inhaled air in the studied area, similar to the nose so that it is possible to comprehensively study the structural components.

Transport functions depend on accurate predictions of the airflow nature. Therefore, the numerical model is tested in an anatomically accurate computational model by comparing simulated velocity profiles with values from the measurement. The basic equations of the incompressible laminar air flow with constant viscosity and thermal conductivity are the continuity, Navier - Stokes, energy conservation and convection - diffusion equations.

The flow behavior during breathing varies depending on environmental conditions. Therefore, three-dimensional computational studies of transport phenomena in the nasal cavity were carried out under different environmental conditions. In all cases, the inhaled air was heated and moistened almost to the state of the nasal tissue before reaching the nasopharynx. Complex geometries increase the rate of local transfer of heat and moisture by improving mixing and maintaining thin boundary layers. A normal nose can withstand various extreme conditions. However, irregularities in the blood supply or wetting of the surface can reduce the flow rate of heat or moisture in the inhaled air. Further research will need to improve detailed descriptions of the heat transfer and humidification dynamics.

It should be noticed that for this study there are several limitations. The first limitation is the grid size since even most powerful computers cannot provide data from the numerical simulation for full three-dimensional cases with all details of the nasal cavity. The second limitation is the complexity of the implementation and analysis of experimental data from the tomographic images for the different nasal sinus. The third limitation is the tomographic apparatus, which gives only a limited number of images.

The proposed 3D model has the potential for use as geometric standard in future computational studies, as well as for use as a reference in diagnostics. The developed procedure can also be used to construct standard nose geometries for various identifiable groups in a larger population.

## Data Availability

The datasets used and/or analyzed during the current study are available from the corresponding author on reasonable request.

## Abbreviations

CFD: Computational fluid dynamics
SIMPLE: Semi-Implicit Method for Pressure Linked Eqs.
CT: Computed tomographic
CAD: Computer-aided design
